# Diholmium(III) tris­ulfate tetra­hydrate. Corrigendum

**DOI:** 10.1107/S1600536812032849

**Published:** 2012-07-28

**Authors:** Wanli Zhou, Shahua Ding, Xin Xu, Yan Xu

**Affiliations:** a Institute of Chemistry for Functionalized Materials, College of Chemistry and Chemical Engineering, Liaoning Normal University, Dalian 116029, People’s Republic of China; b Department of Chemistry, Tonhua Normal University, Tonghua 134002, People’s Republic of China; c Heavy Oil Company, Liaohe Petroleum Filiale, China National Petroleum Corporation (CNPC), Shiyou Street No. 96, Panjin 124010, People’s Republic of China

## Abstract

The title and formula in the paper by Zhou *et al.* [*Acta Cryst.* (2007), E**63**, i194] are corrected.

## Abstract 2

Corrigendum to *Acta Cryst.* (2007), E**63**, i194.

In the paper by Zhou *et al.* (2007) ▶, the chemical name and formula are incorrect with respect to the number of water molecules. The correct title should be ‘Diholmium(III) tri­sulfate octa­hydrate’ and the formula ‘[Ho_2_(SO_4_)_3_(H_2_O)_8_]’. The *Crystal data* in the original paper are correct apart from the formula.
